# Predictive factors for progression‐free survival in non‐small cell lung cancer patients receiving nivolumab based on performance status

**DOI:** 10.1002/cam4.2807

**Published:** 2019-12-27

**Authors:** Yuichi Adachi, Akihiro Tamiya, Yoshihiko Taniguchi, Takatoshi Enomoto, Kouji Azuma, Shunichi Kouno, Yuji Inagaki, Nobuhiko Saijo, Kyoichi Okishio, Shinji Atagi

**Affiliations:** ^1^ Department of Internal Medicine National Hospital Organization Kinki‐Chuo Chest Medical Center Sakai Japan; ^2^ Clinical Research Center National Hospital Organization Kinki‐Chuo Chest Medical Center Sakai Japan

**Keywords:** immunotherapy, nivolumab, non-small cell lung cancer, performance status, prognosis

## Abstract

**Background:**

Nivolumab has promising efficacy for the treatment of non‐small cell lung cancer (NSCLC). Various predictive factors for nivolumab response in those with NSCLC have been reported, including performance status (PS). The objective of this retrospective study was to determine the predictive factors for nivolumab response in those with NSCLC with good PS and those with poor PS.

**Methods:**

We retrospectively collected pretreatment clinical data of 296 consecutive patients with NSCLC treated with nivolumab. We investigated the relationship between progression‐free survival (PFS) and patient characteristics and analyzed predictive factors associated with good PS (PS 0‐1) or poor PS (PS 2‐4).

**Results:**

The median age of patients was 70 years; 206 patients were male, and 224 were classified as having good PS (PS 0‐1). The median PFS was 3.0 months, 3.7 months, and 1.2 months for all patients, patients with good PS, and patients with poor PS respectively. Multivariate analysis showed that never smoking (hazard ratio [HR], 1.77; 95% confidence interval [CI], 1.15‐2.75), high C‐reactive protein (CRP) (HR, 1.39; 95% CI, 1.00‐1.93), liver metastasis (HR, 1.95; 95% CI, 1.24‐3.07), pleural effusion (HR, 1.45; 95% CI, 1.06‐2.00), and steroid use (HR, 2.85; 95% CI, 1.65‐4.94) were associated with significantly shorter PFS in patients with good PS. A high advanced lung cancer inflammation index (ALI) was significantly associated with longer PFS in patients with poor PS (HR, 0.24; 95% CI, 0.08‐0.79).

**Conclusions:**

In patients with NSCLC treated with nivolumab, the factors found to be predictive of shorter PFS in patients with good PS were never smoking, high CRP, liver metastasis, pleural effusion, and steroid administration, whereas high ALI was predictive of longer PFS in patients with poor PS.

## Graphical abstract

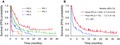

## 1 INTRODUCTION

Immunotherapy has led to great breakthroughs in cancer treatment. Nivolumab, an anti‐programmed death ligand 1 (PD‐L1) antibody, has shown good efficacy for treating patients with non‐small cell lung cancer (NSCLC) as a second or later line treatment. Two phase III studies have reported that the progression‐free survival (PFS) of patients with NSCLC treated with nivolumab was 2.3‐3.5 months; however, the overall response rate (ORR) was only 19%‐20%.^1^, ^2^

Many factors predict the response of patients with NSCLC to nivolumab treatment, including performance status (PS) steroid use at baseline, smoking status, central nervous system metastasis, liver metastasis, and advanced lung inflammatory index (ALI).^3^, ^4^, ^5^, ^6^, ^7^ In clinical practice, there are many patients with NSCLC with poor PS, who are less likely to benefit from nivolumab treatment. Moreover, there are many patients with NSCLC with good PS who may also not benefit from nivolumab treatment. Therefore, it is essential to clarify the predictive factors for response to nivolumab based on PS. Specifically, it is important to clarify factors that can help predict responses to nivolumab in those with poor PS as there are no data from major clinical trials on this topic. The objective of this study was to identify the predictive factors for nivolumab treatment in those with NSCLC with good PS and those with poor PS.

## 2 MATERIALS AND METHODS

### 2.1 Patients

The medical records of 296 consecutive patients with NSCLC treated with nivolumab at our institution between December 17, 2015, and December 31, 2018, were retrospectively reviewed. Study approval was obtained from the appropriate institutional review board (2019‐014).

### 2.2 Data collection

Data collected from the patients included the following: age, gender, Eastern Cooperative Oncology Group (ECOG) PS, histological type, smoking history, body mass index (BMI), epidermal growth factor receptor (EGFR) mutation status, anaplastic lymphoma kinase (ALK) mutation status, number of prior systemic therapies, chest radiation history, laboratory data (C‐reactive protein [CRP], lactate dehydrogenase [LDH], albumin [ALB], neutrophil count, and lymphocyte count), neutrophil to lymphocyte ratio (NLR), ALI, liver metastasis, brain metastasis, pleural effusion, and whether or not steroids were being used at the commencement of nivolumab treatment. The NLR score was calculated as follows: neutrophil counts/ lymphocyte count; the ALI score was calculated as follows: BMI × ALB/NLR.

Responses to nivolumab treatment were determined using the Response Evaluation Criteria in Solid Tumors, version 1.1.^8^ PFS was estimated as the duration between nivolumab treatment initiation and disease progression or death from any cause. We investigated the relationship between PFS after nivolumab treatment and patient characteristics at the time nivolumab treatment began. We also investigated the relationship between overall survival (OS) and patient characteristics. In this study, we regarded the PFS as the primary endpoint for predicting the effects of nivolumab since OS is affected in various ways including those related to pre‐ and post‐treatment. Furthermore, we determined predictive factors in patients with NSCLC treated with nivolumab based on good or poor PS. We defined good PS as a PS of 0‐1 and poor PS as a PS of 2‐4. Patients were followed‐up until February 28, 2019.

### 2.3 Statistical analyses

Based on previous reports, the cut‐off values for BMI, LDH, CRP, NLR, and ALI were defined as 20 kg/m^2^,^9^ 240 IU/L,^10^ 1 mg/dL,^11^ 4,^12^ and 18^13^ respectively. The cut‐offs for other continuous variables were defined using their median values. Statistical analyses were conducted using the JMP statistical software program (14th version, SAS Institute Inc, Cary, NC, USA) to compare PFS based on patient characteristics. A p‐value less than 0.05 was considered statistically significant. Survival curves were generated using the Kaplan‐Meier method, and the differences between the two groups were compared with the log‐rank test. Univariate and multivariate analyses were performed using the Cox proportional hazards model. Only factors with a *P*‐value less than .05 in the univariate analysis were included in the multivariate analysis.

## 3 RESULTS

### 3.1 Patient baseline characteristics

Patient characteristics at the commencement of nivolumab treatment for the entire cohort as well as the PS subgroups are shown in Table 1. In the entire cohort, the median age was 70, and the majority of patients (69.6%) were male. In terms of PS, 224 patients had good PS (PS0, 42; PS1, 182) and 72 patients had poor PS (PS2, 52; PS3, 14; PS4, 6). There were 185 cases of adenocarcinoma, 81 cases of squamous cell carcinoma, and 30 cases with other histologies. Overall, 30 patients used steroids at the start of nivolumab treatment for the following reasons: brain metastasis in 10 patients, fatigue in 8 patients, radiation pneumonitis in 5 patients, autoimmune disorder in 4 patients, and other unspecified factors in 3 patients. When we compared clinical data between those with good PS and those with poor PS, absence of prior chest radiotherapy (*P* = .046), CRP ≥ 1 mg/dL (*P* < .001), NLR ≥ 4 (*P* < .001), ALB < 1 (*P* < .001), ALI < 18 (*P* < .001), liver metastasis (*P* = .032), and steroid use at the start of nivolumab treatment (*P* = .006) were significantly associated with poor PS.

**Table 1:** Patient characteristics at the commencement of nivolumab treatment

	All patients (n = 296)	Good PS (0‐1) (n = 224)	Poor PS (2‐4) (n = 72)	*P*‐value
Age, years, median (IQR)	70 (64‐76)	70 (64‐75)	73.5 (66‐79.8)	.018
Gender, number (%)				.66
Male	206 (69.6)	154 (68.8)	52 (72.2)	
Female	90 (30.4)	70 (31.3)	20 (27.8)	
PS, number (%)				
0	42 (14.2)	42 (18.8)		
1	182 (61.5)	182 (81.3)		
2	52 (17.6)		52 (72.2)	
3	14 (4.7)		14 (19.4)	
4	6 (2.0)		6 (8.3)	
Histology, number (%)				.87
Adenocarcinoma	185 (62.5)	143 (63.8)	42 (58.3)	
Squamous cell carcinoma	81 (27.4)	64 (28.6)	17 (23.6)	
Others	30 (10.1)	17 (7.6)	13 (18.1)	
Smoking status, number (%)				.74
Current or former smoker	238 (80.4)	181 (80.8)	57 (79.2)	
Never	58 (19.6)	43 (19.2)	15 (20.8)	
BMI, kg/m^2^, median (IQR)	21.6 (19.1‐24.1)	21.6 (19.5‐24.2)	21.5 (18.6‐24.1)	.18
*EGFR* mutation, number (%)				.61
Positive	56 (18.9)	41 (18.3)	15 (20.8)	
Negative	176 (59.5)	131 (58.5)	45 (62.5)	
Unknown	64 (21.6)	52 (23.2)	12 (16.7)	
*ALK* mutation, number (%)				>.99
Positive	1 (0.3)	1 (0.4)	0 (0)	
Negative	218 (73.6)	167 (74.6)	51 (70.8)	
Unknown	77 (26.0)	56 (25.0)	21 (29.2)	
Number of prior systemic therapies, number (%)				.38
1	145 (49.0)	107 (47.8)	38 (52.8)	
2	67 (22.6)	57 (25.4)	10 (13.9)	
3	39 (13.2)	27 (12.1)	12 (16.7)	
4	21 (7.1)	15 (6.7)	6 (8.3)	
5	11 (3.7)	8 (3.6)	3 (4.2)	
>5	13 (4.4)	10 (4.5)	3 (4.2)	
Prior chest radiotherapy, number of patients (%)				.046
Yes	62 (20.9)	53 (23.7)	9 (12.5)	
No	234 (79.1)	171 (76.3)	63 (87.5)	
Neut,/μL, median (IQR)	4500 (3386‐6405)	4368 (3324‐5900)	5633 (3425‐9125)	.001
Lymp,/μL, median (IQR)	1230 (991‐1612)	1300 (1000‐1635)	1102 (800‐1589)	.020
CRP, mg/dL, median (IQR)	0.87 (0.26‐3.24)	0.74 (0.21‐2.50)	2.27 (0.59‐7.16)	<.001
LDH, IU/L, median (IQR)	224 (188‐289)	222 (187‐281)	233 (190‐381)	.15
ALB, g/dL, median (IQR)	3.6 (3.2‐4.0)	3.7 (3.3‐4.0)	3.2 (2.6‐3.6)	<.001
NLR, median (IQR)	3.54 (2.45‐6.16)	3.33 (2.24‐5.29)	5.5 (2.95‐8.32)	<.001
ALI, median (IQR)	21.3 (11.9‐34.1)	23.2 (14.2‐38.0)	12.2 (7.4‐25.8)	<.001
Liver metastasis, number (%)				.032
Yes	42 (14.2)	26 (11.6)	16 (22.2)	
No	254 (85.8)	198 (88.4)	56 (77.8)	
Brain metastasis, number (%)				.76
Yes	78 (26.4)	58 (25.9)	20 (27.8)	
No	218 (73.6)	166 (74.1)	52 (72.2)	
Pleural effusion, number (%)				.34
Yes	128 (43.2)	93 (41.5)	35 (48.6)	
No	168 (56.8)	131 (58.5)	37 (51.4)	
Use of systemic steroids at the commencement of nivolumab, number (%)				.006
Yes	30 (10.1)	16 (7.1)	14 (19.4)	
No	266 (89.9)	208 (92.9)	58 (80.6)	

Abbreviations: ALB, albumin; ALI, advanced lung cancer inflammation index; ALK, anaplastic lymphoma kinase; BMI, body mass index; CRP, C‐reactive protein; EGFR, epidermal growth factor receptor; IQR, interquartile range; LDH, lactate dehydrogenase; Lymp, lymphocyte; Neut, neutrophil; NLR, neutrophil to lymphocyte ratio; PS, performance status; PS, performance status.

### 3.2 Response to treatment

**Figure 1:**
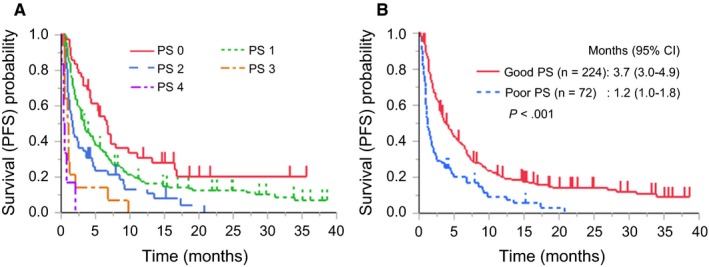
Kaplan‐Meier curves of progression‐free survival (PFS) in patients with non‐small cell lung cancer treated with nivolumab stratified by (A) performance status (PS), and (B) good PS (PS 0 or 1) or poor PS (PS 2‐4)

At the end of the follow‐up period, 253 patients (85.5%) had disease progression and 195 (65.9%) had died. The median follow‐up period was 26.6 months (Kaplan‐Meier estimates). The median PFS of all 296 patients was 3.0 months (95% confidence interval [CI]: 2.4‐3.7). Kaplan‐Meier curves of patients with NSCLC treated with nivolumab based on PS are shown in Figure 1. The median PFS of patients with NSCLC with good PS was 3.7 (95% CI, 3.0‐4.9) months, and the median PFS of patients with NSCLC with poor PS was 1.2 months (95% CI, 1.0‐1.8). There was a significant difference in PFS between patients with NSCLC treated with nivolumab with good PS versus those with poor PS (*P* < .001). The median OS of all patients, patients with good PS, and patients with poor PS was 10.5 (95% CI, 8.3‐12.5) months, 13.4 (95% CI, 10.9‐17.2) months and 3.9 (2.0‐5.7) months respectively (Figure S1). There was a significant difference in OS between patients with NSCLC treated with nivolumab with good PS vs those with poor PS (*P* < .001). Responses to nivolumab treatment in the PS subgroups are shown in Table 2. The ORR and disease control rate (DCR) in the entire cohort were 14.5% and 54.7% respectively. The ORRs of patients with NSCLC with a PS of 0, 1, 2, 3, and 4 were 23.8%, 14.8%, 9.6%, 7.1%, and 0%, respectively; the corresponding DCRs were 71.4%, 59.9%, 40.4%, 14.3%, and 0% respectively. The ORR and DCR consistently decreased with worsening PS in patients with NSCLC treated with nivolumab.

**Table 2:** Response to nivolumab treatment according to PS

	CR	PR	SD	PD	NE	ORR (%)	DCR (%)
All (n = 296)	4	39	119	129	5	14.5	54.7
PS0 (n = 42)	1	9	20	12	0	23.8	71.4
PS1 (n = 182)	3	24	82	69	4	14.8	59.9
PS2 (n = 52)	0	5	16	31	0	9.6	40.4
PS3 (n = 14)	0	1	1	11	1	7.1	14.3
PS4 (n = 6)	0	0	0	6	0	0	0
Good PS (n = 224)^a^	4	33	102	81	4	16.5	62.1
Poor PS (n = 72)^a^	0	6	17	48	1	8.3	31.9

Abbreviations: CR, complete response; DCR, disease control rate; NE, not evaluated; ORR, overall response rate; PD, progressive disease; PR, partial response; PS, performance status; SD, stable disease.

a Good PS and poor PS were defined as a PS of 0 to 1 and a PS of 2 to 4 respectively.

### 3.3 Association of patient characteristics with PFS

The results of the univariate and multivariate analyses of factors associated with PFS in the entire cohort are shown in Table 3. In the univariate analysis, a PS of 2‐4, never smoking, driver mutations including those of *EGFR* and *ALK*, LDH ≥ 240 IU/L, CRP ≥ 1 mg/dL, NLR ≥ 4, liver metastasis, brain metastasis, pleural effusion, and steroid use at the commencement of nivolumab treatment were associated with a shorter PFS. An ALB ≥ 3.5 g/dL and ALI ≥ 18 was associated with a longer PFS. In the multivariate analysis, a PS of 2‐4 (HR, 1.62; 95% CI, 1.19‐2.20), never smoking (HR, 1.68; 95% CI, 1.16‐2.43), driver mutation (HR, 1.45; 95% CI, 1.02‐2.07), CRP ≥ 1 mg/dL (HR, 1.52; 95% CI, 1.10‐2.09), liver metastasis (HR, 1.62; 95% CI, 1.11‐2.36), and steroid use (HR, 2.57; 95% CI, 1.65‐4.01) were significantly associated with a shorter PFS. Regarding OS, multivariate analysis revealed that PS, ALB, NLR, ALI, liver metastasis, and steroid use were the predictive factors of OS (Table S1).

**Table 3:** Univariate and multivariate Cox proportional hazards model analysis of factors associated with progression‐free survival in all patients

	Univariate analysis			Multivariate analysis		
	HR	95% CI	*P*‐value	HR	95% CI	*P*‐value
Female	1.04	0.79‐1.36	.79			
Age <70 y	0.92	0.72‐1.17	.49			
PS 2‐4	2.07	1.56‐2.75	<.001	1.62	1.19‐2.20	.002
Squamous cell carcinoma	1.05	0.79‐1.38	.74			
Never smoking	1.42	1.05‐1.93	.023	1.68	1.16‐2.43	.006
BMI <20 kg/m^2^	1.17	0.90‐1.52	.24			
Driver mutation positivity (*EGFR*, *ALK*)	1.45	1.07‐1.96	.016	1.45	1.02‐2.07	.039
≥ 2 prior treatments	1.19	0.93‐1.52	.18			
Prior chest radiotherapy	0.85	0.63‐1.15	.29			
LDH ≥240 IU/L	1.34	1.05‐1.72	.020	1.10	0.83‐1.45	.52
CRP ≥1 mg/dL	1.57	1.23‐2.02	<.001	1.52	1.10‐2.09	.01
ALB ≥3.5 g/dL	0.64	0.50‐0.83	<.001	0.89	0.66‐1.21	.47
NLR ≥4	1.38	1.07‐1.77	.011	0.69	0.42‐1.12	.13
ALI ≥18	0.60	0.46‐0.77	<.001	0.66	0.39‐1.10	.11
Liver metastasis	2.04	1.44‐2.90	<.001	1.62	1.11‐2.36	.012
Brain metastasis	1.33	1.01‐1.75	.040	1.29	0.96‐1.75	.091
Pleural effusion	1.33	1.04‐1.71	.023	1.29	0.98‐1.70	.075
Use of steroids	2.45	1.64‐3.66	<.001	2.57	1.65‐4.01	<.001

Abbreviations: ALB, albumin; ALI, advanced lung cancer inflammation index; ALK, anaplastic lymphoma kinase; BMI, body mass index; CI, confidence interval; CRP, C‐reactive protein; EGFR, epidermal growth factor receptor; HR, hazard ratio; LDH, lactate dehydrogenase; NLR, neutrophil to lymphocyte ratio; PS, performance status.

### 3.4 Association of patient characteristics with PFS in PS subgroups

PS was a significant predictive factor of PFS in the entire cohort of patients with NSCLC treated with nivolumab. We also investigated the factors predictive of PFS in these patients based on PS. The results of the univariate and multivariate analyses of factors associated with PFS in those with good PS and those with poor PS are shown in Tables 4 and 5. Among nivolumab‐treated patients with NSCLC with good PS, the univariate analysis revealed that smoking status, driver mutation, LDH, CRP, liver metastasis, pleural effusion, and steroid use were significantly associated with PFS. Multivariate analysis revealed that never smoking (HR, 1.77; 95% CI, 1.15‐2.75), CRP ≥ 1 mg/dL (HR, 1.39; 95% CI, 1.00‐1.93), liver metastasis (HR, 1.95; 95% CI, 1.24‐3.07), pleural effusion (HR, 1.45; 95% CI, 1.06‐2.00), and steroid use (HR, 2.85; 95% CI, 1.65‐4.94) were significantly associated with a shorter PFS among patients with NSCLC treated with nivolumab with good PS. Regarding OS, the multivariate analysis revealed that a low ALB, liver metastasis, pleural effusion, and steroid use were significantly associated with a shorter OS among patients treated with nivolumab with good PS (Table S2).

**Table 4:** Univariate and multivariate Cox proportional hazards model analysis of factors associated with progression‐free survival in patients with good PS

	Univariate analysis			Multivariate analysis		
	HR	95% CI	*P*‐value	HR	95% CI	*P*‐value
Female	1.14	0.84‐1.55	.41			
Age <70 y	0.84	0.63‐1.13	.25			
Squamous cell carcinoma	1.02	0.75‐1.41	.88			
Never smoking	1.48	1.03‐2.11	.031	1.77	1.15‐2.75	.01
BMI <20 kg/m^2^	1.10	0.80‐1.51	.55			
Driver mutation positivity (*EGFR*, *ALK*)	1.45	1.05‐2.12	.025	1.37	0.92‐2.02	.12
≥2 prior treatments	1.21	0.90‐1.61	.20			
Prior chest radiotherapy	0.93	0.66‐1.31	.68			
LDH ≥240 IU/L	1.38	1.03‐1.85	.029	1.13	0.84‐1.53	.43
CRP ≥1 mg/dL	1.35	1.02‐1.81	.039	1.39	1.00‐1.93	.048
ALB ≥3.5 g/dL	0.82	0.60‐1.12	.20			
NLR ≥4	1.13	0.84‐1.52	.41			
ALI ≥18	0.75	0.55‐1.02	.065			
Liver metastasis	2.15	1.39‐3.34	<.001	1.95	1.24‐3.07	.004
Brain metastasis	1.36	0.99‐1.87	.060			
Pleural effusion	1.35	1.01‐1.80	.044	1.45	1.06‐2.00	.021
Steroid use	2.18	1.28‐3.72	.004	2.85	1.65‐4.94	<.001

Abbreviations: ALB, albumin; ALI, advanced lung cancer inflammation index; ALK, anaplastic lymphoma kinase; BMI, body mass index; CI, confidence interval; CRP, C‐reactive protein; EGFR, epidermal growth factor receptor; HR, hazard ratio; LDH, lactate dehydrogenase; NLR, neutrophil to lymphocyte ratio; PS, performance status.

**Table 5:** Univariate and multivariate Cox proportional hazards model analysis of factors associated with progression‐free survival in patients with poor PS

	Univariate analysis			Multivariate analysis		
	HR	95% CI	*P*‐value	HR	95% CI	*P*‐value
Female	0.82	0.48‐1.42	.48			
Age <70 y	1.58	0.96‐2.60	.070			
Squamous cell carcinoma	0.67	0.37‐1.22	.19			
Never smoking	1.38	0.76‐2.51	.29			
BMI < 20 kg/m^2^	1.07	0.66‐1.75	.77			
Driver mutation positivity (*EGFR*, *ALK*)	1.36	0.75‐2.46	.32			
≥2 prior treatments	1.19	0.73‐1.94	.48			
Prior chest radiotherapy	0.73	0.36‐1.48	.38			
LDH ≥240 IU/L	1.14	0.70‐1.86	.59			
CRP ≥1 mg/dL	2.03	1.21‐3.40	.008	1.40	0.75‐2.61	.29
ALB ≥3.5 g/dL	0.55	0.32‐0.97	.038	0.72	0.39‐1.34	.31
NLR ≥4	1.89	1.14‐3.13	.014	0.40	0.13‐1.21	.11
ALI ≥18	0.41	0.24‐0.70	.001	0.24	0.08‐0.79	.018
Liver metastasis	1.39	0.77‐2.51	.27			
Brain metastasis	1.18	0.69‐2.03	.54			
Pleural effusion	1.10	0.68‐1.80	.69			
Steroid use	1.93	1.02‐3.64	.044	1.66	0.84‐3.29	.14

Abbreviations: ALB, albumin; ALI, advanced lung cancer inflammation index; ALK, anaplastic lymphoma kinase; BMI, body mass index; CI, confidence interval; CRP, C‐reactive protein; EGFR, epidermal growth factor receptor; HR, hazard ratio; LDH, lactate dehydrogenase; NLR, neutrophil to lymphocyte ratio; PS, performance status.

For those with poor PS, the univariate analysis revealed that CRP, ALB, NLR, ALI, and steroid use were significantly associated with PFS. Multivariate analysis revealed that ALI ≥ 18 (HR, 0.24; 95% CI, 0.08‐0.79) was independently associated with a longer PFS in patients with NSCLC treated with nivolumab with poor PS. We did not identify statistically significant predictive factors of OS among nivolumab‐treated patients with poor PS (Table S3). However, there was a trend showing an association between high ALI and longer OS.

## 4 DISCUSSION

We found that the PS was strongly associated with PFS in patients with NSCLC receiving nivolumab treatment. We also found that predictive factors for nivolumab response were different between patients with NSCLC with good PS and those with poor PS. Never smoking, CRP ≥ 1 mg/dL, liver metastasis, pleural effusion, and steroid use were predictive of shorter PFS in patients with NSCLC treated with nivolumab with good PS, whereas ALI ≥ 18 was predictive of longer PFS in patients with NSCLC treated with nivolumab with poor PS. A similar trend was seen for OS. Our results suggest that these factors may serve as predictors of response to nivolumab in patients with NSCLC with differing PS.

PS is the most essential factor in managing lung cancer. Before the immunotherapy era, clinical trials evaluating chemotherapy in patients with NSCLC stratified them into those with a PS of 0‐1 and those with a PS of 2.^14^ These clinical trials revealed that PS is the most powerful independent predictive factor for chemotherapy response in patients with advanced NSCLC. Recently, several studies revealed that PS is also a strong independent factor for immune therapy in the treatment of NSCLC.^4^, ^15^, ^16^ In the clinical setting, patients with cancer often have a poor PS, and these patients represent a heterogeneous population. The definition of PS does not take into account various factors including age, tumor mutation burden, and comorbidities. Our results showed that inflammation markers, such as CRP, ALB, NLR, and ALI, were significantly associated with PS. Overall these results showed that systemic Inflammation may lead to worse PS.

Immune checkpoint inhibitors play an important role in managing NSCLC; however, the ORR for nivolumab monotherapy is only 19%‐20%, and patients who can benefit from nivolumab are limited.^1^, ^2^, ^17^, ^18^ Therefore, it is necessary to determine factors that predict response to nivolumab in patients with NSCLC. Many predictive factors for nivolumab treatment in patients with NSCLC have previously been reported. Our results showed that PS was significantly associated with PFS in patients with NSCLC receiving nivolumab treatment. Additionally, never smoking, driver mutation positivity, high CRP, liver metastasis, and steroid use at the commencement of nivolumab treatment were associated with worse PFS in nivolumab‐treated patients. These predictive factors for nivolumab response in patients with NSCLC, including PS, are consistent with those identified in previous reports.^3^, ^10^, ^19^, ^20^, ^21^, ^22^, ^23^, ^24^

The factors associated with PFS in nivolumab‐treated patients with NSCLC with good PS were similar to those identified in the entire cohort. Smoking has been reported to enhance tumor mutation burden; consistent with this finding, nivolumab is efficacious in smokers.^25^ Previous studies reported that steroid use, especially for palliative indications, was a predictive factor of worse outcome in patients with NSCLC treated with immune checkpoint inhibitors. In our study, steroid was mostly used in situations associated with palliative treatment. We confirmed steroid use was a worse predictive factor of PFS in patients with NSCLC treated with nivolumab.^3^, ^24^ Consistent with our findings, liver metastasis and pleural effusion have also been reported as factors predictive of nivolumab efficacy in patients with NSCLC.^21^

In patients with NSCLC treated with nivolumab who had poor PS, ALI was found to be an independent predictor of PFS. ALI has previously been reported to be a significant independent predictor of response to nivolumab.^13^ The ALI was devised to assess the degree of systemic inflammation in patients with advanced NSCLC.^26^ Combined with the BMI, serum ALB, and NLR, the ALI serves as a more comprehensive indicator of systemic inflammation and therefore may be an important predictor of efficacy in patients treated with nivolumab.

The predictive factors were different between patients with good PS and poor PS. This difference may be due to the heterogeneity among patients with poor PS. In our study, the median ALI was lower in patients with NSCLC treated with nivolumab with poor PS than in those with good PS. Previous studies also reported that systemic inflammation was associated with PS.^27^ For some patients, the PS was reduced due to comorbidities even when their systemic inflammation status was relatively preserved. Considering the heterogeneity of patients who received nivolumab‐treatment with poor PS, whose systemic inflammation status was worse than patients with good PS, preserved inflammation status considered to be stronger predictor in patients with poor PS than with good PS. This result suggests that patients with NSCLC with poor PS with a high ALI could benefit from nivolumab treatment.

Although the current study presents findings that have clinical implications, there are several limitations. First, this study is retrospective, and the data are from a single institute. However, the number of patients in this study was relatively high. Second, PD‐L1 expression status could not be assessed as a predictive factor because of the lack of routine PD‐L1 testing. Measurement of PD‐L1 expression status is not required for nivolumab treatment in our country. Third, although OS data were obtained, it was difficult to assess the effect of nivolumab on OS because the time from diagnosis to nivolumab treatment varied depending on the patients. Finally, the number of patients with NSCLC treated with nivolumab with poor PS was small. However, to the best of our knowledge, this study was the first report on predictive factors of nivolumab treatment in patients with NSCLC with poor PS. Patients with poor PS are commonly excluded from clinical trials, and therefore data on the response of patients with poor PS are unlikely to be reported. Our finding that ALI is a predictive factor for patients with NSCLC with poor PS receiving nivolumab has clinical significance.

## 5 CONCLUSIONS

PS was associated with PFS in patients with NSCLC receiving nivolumab treatment. Never smoking, high CRP, liver metastasis, pleural effusion, and steroid use at the commencement of nivolumab treatment were predictive of worse PFS in patients with NSCLC who received nivolumab treatment with good PS, and a high ALI was predictive of better PFS in patients with poor PS.

## Data Availability

Research data are not shared.
