# Supplementary material for: Predictive factors for progression‐free survival in non‐small cell lung cancer patients receiving nivolumab based on performance status
Source: Cancer Med. 2019 Dec 27;9(4):1383–91. doi: 10.1002/cam4.2807 (PMC7013052; doi:10.1002/cam4.2807)
Supplement: Supplementary file 1 [file CAM4-9-1383-s001.pptx]

## Slide 1
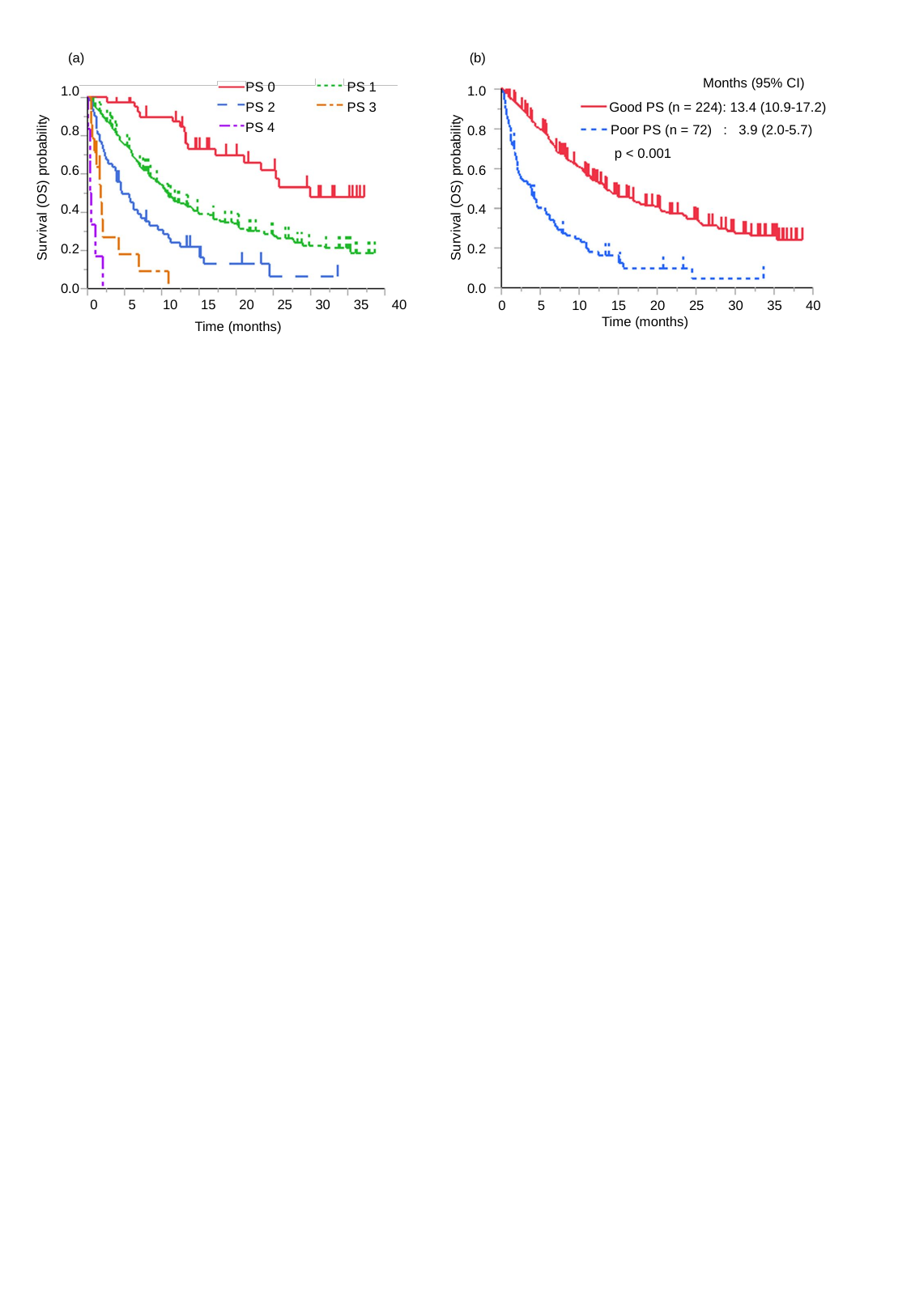

(a)
(b)
Months (95% CI)
PS 0
PS 1
1.0
1.0
PS 2
PS 3
Good PS (n = 224): 13.4 (10.9-17.2)
PS 4
Poor PS (n = 72) : 3.9 (2.0-5.7)
0.8
0.8
p < 0.001
0.6
0.6
Survival (OS) probability
Survival (OS) probability
0.4
0.4
0.2
0.2
0.0
0.0
0
5
10
15
20
25
30
35
40
0
5
10
15
20
25
30
35
40
Time (months)
Time (months)
