# Supplementary material for: Predictive factors for progression‐free survival in non‐small cell lung cancer patients receiving nivolumab based on performance status
Source: Cancer Med. 2019 Dec 27;9(4):1383–91. doi: 10.1002/cam4.2807 (PMC7013052; doi:10.1002/cam4.2807)
Supplement: Supplementary file 2 [file CAM4-9-1383-s002.pptx]

## Slide 1
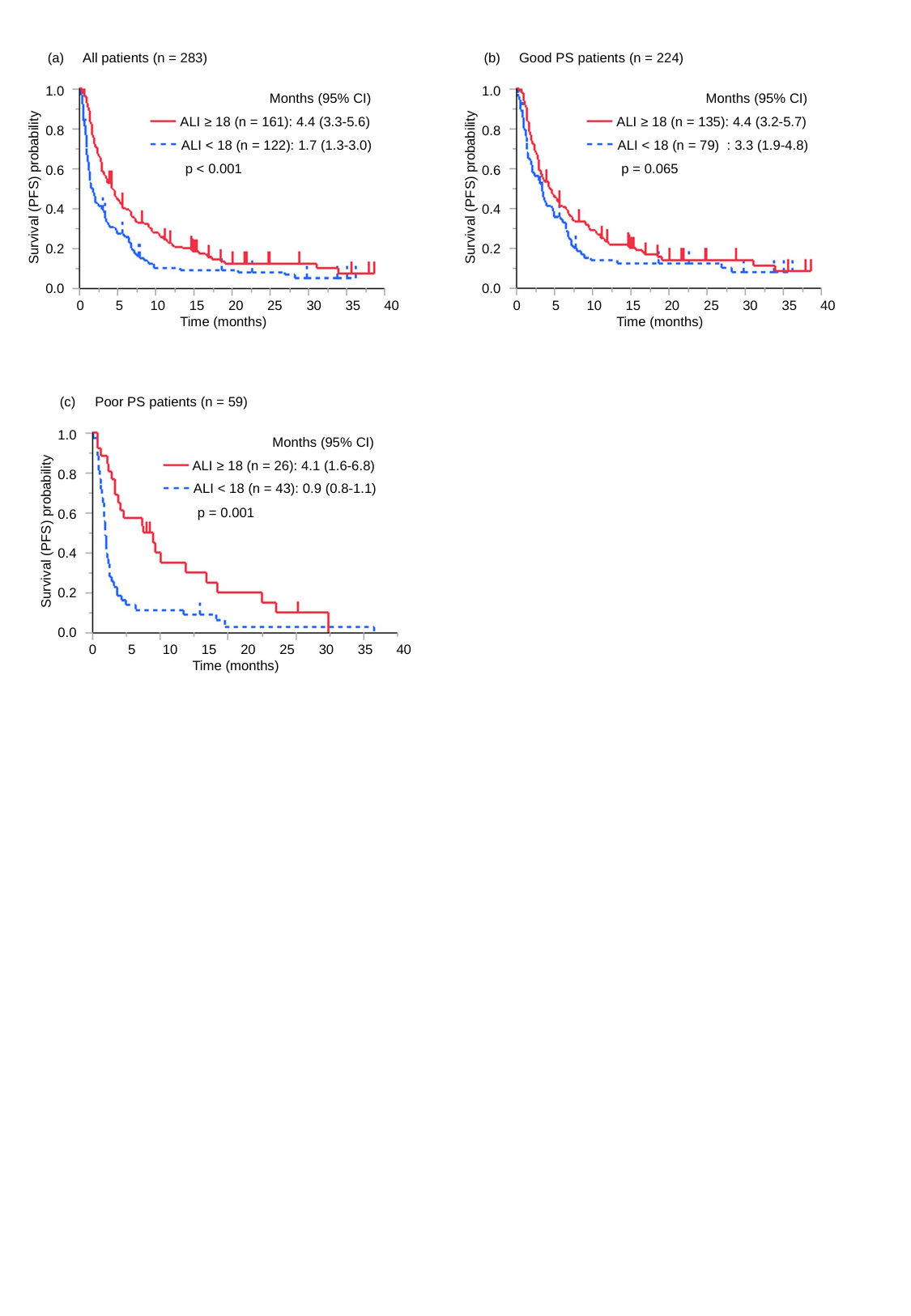

(a)
All patients (n = 283)
1.0
Months (95% CI)
ALI ≥ 18 (n = 161): 4.4 (3.3-5.6)
0.8
ALI < 18 (n = 122): 1.7 (1.3-3.0)
p < 0.001
0.6
Survival (PFS) probability
0.4
0.2
0.0
0
5
10
15
20
25
30
35
40
Time (months)
(b)
Good PS patients (n = 224)
1.0
Months (95% CI)
ALI ≥ 18 (n = 135): 4.4 (3.2-5.7)
0.8
ALI < 18 (n = 79) : 3.3 (1.9-4.8)
p = 0.065
0.6
Survival (PFS) probability
0.4
0.2
0.0
0
5
10
15
20
25
30
35
40
Time (months)
(c)
Poor PS patients (n = 59)
1.0
Months (95% CI)
ALI ≥ 18 (n = 26): 4.1 (1.6-6.8)
0.8
ALI < 18 (n = 43): 0.9 (0.8-1.1)
p = 0.001
0.6
Survival (PFS) probability
0.4
0.2
0.0
0
5
10
15
20
25
30
35
40
Time (months)
