# Supplementary material for: Predictive factors for progression‐free survival in non‐small cell lung cancer patients receiving nivolumab based on performance status
Source: Cancer Med. 2019 Dec 27;9(4):1383–91. doi: 10.1002/cam4.2807 (PMC7013052; doi:10.1002/cam4.2807)
Supplement: Supplementary file 3 [file CAM4-9-1383-s003.docx]

**SUPPLEMENTARY TABLE 1.** Univariate and multivariate Cox proportional hazards model analysis of factors associated with overall survival in all patients

|  | Univariate analysis | | |  | Multivariate analysis | | |
| --- | --- | --- | --- | --- | --- | --- | --- |
|  | HR | 95% CI | p-value |  | HR | 95% CI | p-value |
| Female | 0.91 | 0.67-1.24 | 0.56 |  |  |  |  |
| Age < 70 years | 0.83 | 0.63-1.10 | 0.20 |  |  |  |  |
| PS 2-4 | 2.99 | 2.19-4.08 | < 0.001 |  | 2.07 | 1.47-2.91 | < 0.001 |
| Squamous cell carcinoma | 1.17 | 0.85-1.61 | 0.34 |  |  |  |  |
| Never smoking | 1.18 | 0.84-1.67 | 0.34 |  |  |  |  |
| BMI < 20 kg/m^2^ | 1.33 | 0.99-1.78 | 0.055 |  |  |  |  |
| Driver mutation positivity  (*EGFR*, *ALK*) | 1.28 | 0.91-1.80 | 0.15 |  |  |  |  |
| ≥ 2 prior treatments | 1.21 | 0.91-1.60 | 0.19 |  |  |  |  |
| Prior chest radiotherapy | 0.75 | 0.53-1.08 | 0.13 |  |  |  |  |
| LDH ≥ 240 IU/L | 1.36 | 1.03-1.80 | 0.031 |  | 1.14 | 0.83-1.55 | 0.42 |
| CRP ≥ 1 mg/dL | 2.10 | 1.58-2.79 | < 0.001 |  | 1.32 | 0.92-1.91 | 0.12 |
| ALB ≥ 3.5 g/dL | 0.39 | 0.29-0.52 | < 0.001 |  | 0.63 | 0.45-0.92 | 0.015 |
| NLR ≥ 4 | 1.58 | 1.19-2.09 | 0.002 |  | 0.54 | 0.30-0.95 | 0.034 |
| ALI ≥ 18 | 0.47 | 0.35-0.62 | < 0.001 |  | 0.49 | 0.27-0.89 | 0.019 |
| Liver metastasis | 2.12 | 1.45-3.09 | < 0.001 |  | 1.62 | 1.09-2.41 | 0.017 |
| Brain metastasis | 1.31 | 0.96-1.78 | 0.085 |  |  |  |  |
| Pleural effusion | 1.62 | 1.22-2.14 | < 0.001 |  | 1.33 | 0.98-1.81 | 0.069 |
| Use of steroids | 2.28 | 1.50-3.48 | < 0.001 |  | 2.00 | 1.26-3.17 | 0.003 |

Abbreviations: PS, performance status; BMI, body mass index; EGFR, epidermal growth factor receptor; ALK, anaplastic lymphoma kinase; LDH, lactate dehydrogenase; CRP, C-reactive protein; ALB, albumin; NLR, neutrophil to lymphocyte ratio; ALI, advanced lung cancer inflammation index; HR, hazard ratio; CI, confidence interval.

**SUPPLEMENTARY TABLE 2.** Univariate and multivariate Cox proportional hazards model analysis of factors associated with overall survival in patients with good PS

|  | Univariate analysis | | |  | Multivariate analysis | | |
| --- | --- | --- | --- | --- | --- | --- | --- |
|  | HR | 95% CI | p-value |  | HR | 95% CI | p-value |
| Female | 0.98 | 0.69-1.41 | 0.93 |  |  |  |  |
| Age < 70 years | 0.75 | 0.53-1.06 | 0.10 |  |  |  |  |
| Squamous cell carcinoma | 1.18 | 0.80-1.72 | 0.41 |  |  |  |  |
| Never smoking | 1.11 | 0.73-1.68 | 0.62 |  |  |  |  |
| BMI < 20 kg/m^2^ | 1.17 | 0.81-1.69 | 0.39 |  |  |  |  |
| Driver mutation positivity  (*EGFR*, *ALK*) | 1.47 | 0.98-2.21 | 0.057 |  |  |  |  |
| ≥ 2 prior treatments | 1.31 | 0.93-1.85 | 0.12 |  |  |  |  |
| Prior chest radiotherapy | 0.96 | 0.65-1.43 | 0.85 |  |  |  |  |
| LDH ≥ 240 IU/L | 1.40 | 0.99-1.97 | 0.052 |  |  |  |  |
| CRP ≥ 1 mg/dL | 1.95 | 1.39-2.74 | < 0.001 |  | 1.27 | 0.82-1.97 | 0.28 |
| ALB ≥ 3.5 g/dL | 0.45 | 0.31-0.64 | < 0.001 |  | 0.63 | 0.41-0.98 | 0.042 |
| NLR ≥ 4 | 1.30 | 0.92-1.83 | 0.13 |  |  |  |  |
| ALI ≥ 18 | 0.56 | 0.39-0.80 | 0.0012 |  | 0.87 | 0.58-1.32 | 0.52 |
| Liver metastasis | 2.18 | 1.36-3.51 | 0.0013 |  | 1.72 | 1.04-2.85 | 0.034 |
| Brain metastasis | 1.34 | 0.92-1.94 | 0.12 |  |  |  |  |
| Pleural effusion | 1.80 | 1.28-2.53 | < 0.001 |  | 1.67 | 1.16-2.40 | 0.006 |
| Steroid use | 2.01 | 1.13-3.56 | 0.017 |  | 2.14 | 1.16-3.93 | 0.015 |

Abbreviations: PS, performance status; BMI, body mass index; EGFR, epidermal growth factor receptor; ALK, anaplastic lymphoma kinase; LDH, lactate dehydrogenase; CRP, C-reactive protein; ALB, albumin; NLR, neutrophil to lymphocyte ratio; ALI, advanced lung cancer inflammation index; HR, hazard ratio; CI, confidence interval.

**SUPPLEMENTARY TABLE 3.** Univariate and multivariate Cox proportional hazards model analysis of factors associated with overall survival in patients with poor PS

|  | Univariate analysis | | |  | Multivariate analysis | | |
| --- | --- | --- | --- | --- | --- | --- | --- |
|  | HR | 95% CI | p-value |  | HR | 95% CI | p-value |
| Female | 0.86 | 0.48-1.54 | 0.60 |  |  |  |  |
| Age < 70 years | 1.50 | 0.89-2.53 | 0.13 |  |  |  |  |
| Squamous cell carcinoma | 1.66 | 0.92-3.01 | 0.092 |  |  |  |  |
| Never smoking | 1.81 | 0.97-3.37 | 0.062 |  |  |  |  |
| BMI < 20 kg/m^2^ | 1.18 | 0.71-1.95 | 0.52 |  |  |  |  |
| Driver mutation positivity  (*EGFR*, *ALK*) | 0.88 | 0.46-1.66 | 0.69 |  |  |  |  |
| ≥ 2 prior treatments | 1.09 | 0.66-1.81 | 0.74 |  |  |  |  |
| Prior chest radiotherapy | 0.38 | 0.15-0.95 | 0.038 |  | 0.70 | 0.25-1.95 | 0.49 |
| LDH ≥ 240 IU/L | 1.21 | 0.73-2.02 | 0.46 |  |  |  |  |
| CRP ≥ 1 mg/dL | 1.88 | 1.09-3.26 | 0.023 |  | 1.36 | 0.71-2.60 | 0.35 |
| ALB ≥ 3.5 g/dL | 0.55 | 0.30-0.99 | 0.046 |  | 0.75 | 0.40-1.43 | 0.38 |
| NLR ≥ 4 | 1.79 | 1.05-3.06 | 0.032 |  | 0.59 | 0.19-1.79 | 0.35 |
| ALI ≥ 18 | 0.43 | 0.25-0.76 | 0.004 |  | 0.36 | 0.11-1.23 | 0.10 |
| Liver metastasis | 1.31 | 0.71-2.44 | 0.39 |  |  |  |  |
| Brain metastasis | 1.15 | 0.66-1.99 | 0.63 |  |  |  |  |
| Pleural effusion | 1.00 | 0.60-1.67 | 0.99 |  |  |  |  |
| Steroid use | 1.74 | 0.91-3.34 | 0.094 |  |  |  |  |

Abbreviations: PS, performance status; BMI, body mass index; EGFR, epidermal growth factor receptor; ALK, anaplastic lymphoma kinase; LDH, lactate dehydrogenase; CRP, C-reactive protein; ALB, albumin; NLR, neutrophil to lymphocyte ratio; ALI, advanced lung cancer inflammation index; HR, hazard ratio; CI, confidence interval.

**Figure legends**

Figure 1. Kaplan-Meier curves of progression-free survival (PFS) in patients with non-small cell lung cancer treated with nivolumab stratified by (a) performance status (PS), and (b) good PS (PS 0 or 1) or poor PS (PS 2-4).

Supplementary Figure 1. Kaplan-Meier curves of overall survival (OS) in patients with non-small cell lung cancer treated with nivolumab stratified by (a) performance status (PS), and (b) good PS (PS 0 or 1) or poor PS (PS 2-4).
